# *Notes from the Field: *An Outbreak of* Salmonella *Typhimurium Associated with Playground Sand in a Preschool Setting — Madrid, Spain, September–October 2016

**DOI:** 10.15585/mmwr.mm6609a3

**Published:** 2017-03-10

**Authors:** Carmen Olmedo Lucerón, Ana Pérez Meixeira, Isabel Abad Sanz, Victoria Cid Deleyto, Silvia Herrera León, Leonor Gutierrez Ruiz

**Affiliations:** ^1^Area 10 Public Health, General Directorate of Public Health, Health Authority of the Autonomous Community of Madrid, Spain; ^2^National Center of Microbiology, Carlos III Institute of Health, Madrid, Spain.

On September 23, 2016, a gastroenteritis outbreak among young children in a preschool and primary education center located in Getafe, a city in the southern part of the Madrid metropolitan area, was reported to the Community of Madrid Public Health Services. The first five cases occurred on September 14 and affected children aged 3–5 years, who developed symptoms after attending school. An epidemiologic investigation was initiated and included clinical investigation of the identified cases, an active search for additional cases based on school absences, and an environmental assessment.

Three hundred children aged 3–5 years attend the preschool, with classes of approximately 25 students, and the preschool area is separated from the primary school area. A total of 24 cases of gastroenteritis (defined as at least one of four signs or symptoms [fever, diarrhea, abdominal pain, and vomiting] and microbiological confirmation or epidemiologic link to a confirmed case) were identified with symptom onset from September 12 to October 19, 2016 (Figure). Three children were hospitalized and fully recovered. Among the 24 patients, six (25%) were aged 3 years, five (21%) were aged 4 years, and 13 (54%) were aged 5 years. Fifteen cases (63%) occurred in boys. Eighteen (75%) of the affected children used the school meal service and six (25%) did not. Stool specimens were obtained from 17 (71%) affected children; all were positive for non-Typhi *Salmonella* (confirmed cases). Three confirmed cases occurred among six children who did not use the school meal service and 14 occurred among 18 children who did use the meal service. Seven other patients had an epidemiologic link to a laboratory-confirmed case of *Salmonella* and were classified as probable cases. The attack rate for probable and confirmed cases was 8% (24/300). The *Salmonella* isolates were sent to the National Center of Microbiology Reference Laboratory, Carlos III Institute of Health (National Laboratory) for characterization. All isolates were serotyped and found to be *Salmonella* serotype Typhimurium 4,12:i:1,2 (var. Copenhagen). This serotype is widely distributed and associated with foodborne illness, and has been shown to carry a variety of antibiotic resistance genes (*1*).

**FIGURE:**
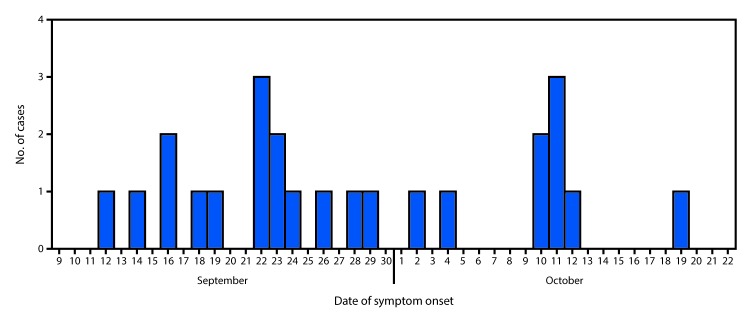
Date of symptom onset among 24 children with *Salmonella* Typhimurium gastroenteritis associated with playground sand — Madrid, Spain, September 12–October 19, 2016

On the day the outbreak was reported, the facilities were inspected, and meal service personnel were interviewed. Samples of potable water and available food prepared during the 2 days before first symptoms began were collected and analyzed; no pathogens were detected. The epidemic curve suggested an ongoing common source (Figure). The only recognized common exposures were attending the early childhood education section of the school, which included children aged 3–5 years, and use of the school playground. No animals were kept at the school. School management hypothesized that the school’s playground, which contains all of the school’s playground equipment, might have become contaminated with animal feces. This area is quite large and covered with loose sand, and contains numerous trees, in which birds roost. On October 10 and November 3, samples of sand from five playground locations were collected for analysis. One sample collected on each date grew *Salmonella* of the same serotype as that identified from the infected children. Both positive samples corresponded to the area of the playground containing swings, seesaws, and slides.

The phagetype (195), the pulsetype (XbaI.0145), and the resistance to tetracycline identified in all isolates is uncommon in humans and was identified in only 4.2% of all isolates typed at the National Laboratory during 2016. This strain has been identified at the National Laboratory from wild and domestic birds (personal communication, S. Herrera, National Laboratory, January 2017). The temporal distribution and microbiologic results suggest that the most likely cause was contact with playground sand contaminated with feces from birds that usually nest in trees above the playground.

Cleaning, sanitation, and structural measures were recommended, and consisted of closing the contaminated areas and renovating the entire playground area. No new cases have been reported since these actions were taken. Long-term control will require a comprehensive strategy that includes environmental interventions and bird population control.

The findings in this report are subject to at least two limitations. First, person-to-person transmission might have occurred. Second, there might have been an underestimation of cases, which can occur in any outbreak investigation. However, these findings support other studies that have identified playground sand as an animal-human interface that facilitates transmission of *Salmonella* (*2*,*3*), particularly among children, and highlight the necessity for enforcement of guidelines to prevent contamination in playground sand and infections among young children (*4*).
